# Fatal encephalitis and myocarditis in young domestic geese (Anser anser domesticus) caused by West Nile virus.

**DOI:** 10.3201/eid0704.010429

**Published:** 2001

**Authors:** D E Swayne, J R Beck, C S Smith, W J Shieh, S R Zaki

**Affiliations:** U.S. Department of Agriculture, Agricultural Research Service, Southeast Poultry Research Laboratory, 934 College Station Road, Athens, GA 30605, USA. dswayne@seprl.usda.gov

## Abstract

During 1999 and 2000, a disease outbreak of West Nile (WN) virus occurred in humans, horses, and wild and zoological birds in the northeastern USA. In our experiments, WN virus infection of young domestic geese (Anser anser domesticus) caused depression, weight loss, torticollis, opisthotonus, and death with accompanying encephalitis and myocarditis. Based on this experimental study and a field outbreak in Israel, WN virus is a disease threat to young goslings and viremia levels are potentially sufficient to infect mosquitoes and transmit WN virus to other animal species.


### Fatal Encephalitis and Myocarditis in Young Domestic Geese (*Anser anser domesticus*) Caused by West Nile Virus

**David E. Swayne,* Joan R. Beck,* Calandra S. Smith,† Wun-Ju Shieh,† and Sharif R. Zaki†**
*U.S. Department of Agriculture, Agricultural Research Service, Southeast Poultry Research Laboratory, Athens, Georgia, USA; †Centers for Disease Control and Prevention, Atlanta, Georgia, USA

During 1999 and 2000, a disease outbreak of West Nile (WN) virus occurred in humans, horses, and wild and zoological birds in the northeastern USA. In our experiments, WN virus infection of young domestic geese (*Anser anser domesticus*) caused depression, weight loss, torticollis, opisthotonus, and death with accompanying encephalitis and myocarditis. Based on this experimental study and a field outbreak in Israel, WN virus is a disease threat to young goslings and viremia levels are potentially sufficient to infect mosquitoes and transmit WN virus to other animal species.

West Nile (WN) virus belongs to the family *Flaviviridae* in the Japanese encephalitis (JE) serocomplex group and is transmitted through various species of adult *Culex* mosquitos to a variety of mammals and birds (1). WN virus was not recognized in North America until the fall of 1999, when an epizootic began with the death of a wild American Crow (*Corvus brachyrhynchos*) in New York (2); an additional 194 deaths in wild birds were confirmed with WN virus infections that fall (2). Most birds that died were from the order Passeriformes; corvids (crows and jays) accounted for >80% of deaths (1). Simultaneously, WN virus emerged as a cause of 62 cases of encephalitis with seven fatalities in humans (3) and 25 cases of neurologic disease in horses in New York City or on Long Island (1).

WN virus has not affected commercial chickens (*Gallus gallus domesticus*) or turkeys (*Meleagridis gallopavo*), which are predominantly raised indoors with low potential for exposure to mosquito vectors (2). Furthermore, experimental studies in chickens and turkeys inoculated subcutaneously with a New York WN virus isolate had low viremia titers and no clinical disease (4,5). However, natural WN virus infections were associated with severe neurologic signs and death in 160 of 400 8- to 10-week-old domestic geese from a flock in Israel (6). The role of domestic geese as a WN virus reservoir in the Israel outbreak is unknown, but goose infection rates in the Sindbis District of the northern Nile Valley were 27%, similar to rates in buffed-back herons (*Bubulcus ibis ibis*), doves (*Streptopelia senegalensis senegalensis*), and domesticated pigeons (*Columbia livia*) and twice the rate in domesticated chickens and ducks (*Anas platyrhynchos*), suggesting that geese may have a role in local WN virus ecology (7). The U.S. WN virus is closely related to WN virus isolates obtained from humans with encephalitis in Romania (1996) and geese in Israel (1998) (8,9). We report the experimental reproduction of neurologic disease and death in young domestic geese with a WN virus isolated from an American Crow on Long Island.

Address for correspondence: David E. Swayne, USDA, ARS, SEPRL, 934 College Station Road, Athens, GA 30605, USA; fax: 706-546-3161; e-mail: dswayne@seprl.usda.gov

### The Study

Four 2-week-old Embden geese were needle-inoculated subcutaneously with $10^{3.3}$ mean tissue culture infective doses ($TCID_{50}$) of Vero cell culture propagated WN virus strain 9/99. A similar dosage was used in previous pathogenesis experiments to infect chickens and turkeys (4,5). The goslings were housed in a Biosafety Level 3 agriculture facility (10). Two uninoculated goslings were maintained in contact with the WN virus-inoculated group and two sham-inoculated controls were maintained in isolation.

During the 21-day study, the four WN virus-inoculated geese lost weight, had decreased activity, and were depressed. WN virus-inoculated gosling #80 had intermittent torticollis and opisthotonus and rhythmic side-to-side movement of the head (Figure) and was euthanized 10 days postinoculation (PI) for persistent neurologic signs. Goslings #6 and #86 died 5 and 6 days PI, respectively (Table). On postmortem examination, gosling #86 had subcutaneous hemorrhage around the joints, pale lungs, and petechial hemorrhages in the splenic capsule. Gosling #80 was dehydrated, lacked abdominal body fat, and had a pale beak, enlarged gall bladder, severe thymic and cloacal bursa atrophy, and excess cerebrospinal fluid. All remaining goslings were euthanized 21 days PI and lacked lesions. Goslings #6 and #86 had the most severe histologic lesions, including moderate nonsuppurative meningoencephalitis (Figure) with occasional apoptotic astrocytes, severe diffuse heterophilic (purulent) to lymphocytic myocarditis (Figure) with edema and myocyte necrosis, and vacuolation and apoptosis of pancreatic acinar cells. Gosling #80 had severe meningoencephalitis with necrosis of astrocytes and microglial cells, edema, and microgliosis; lymphohistiocytic choroiditis, uveitis, and from

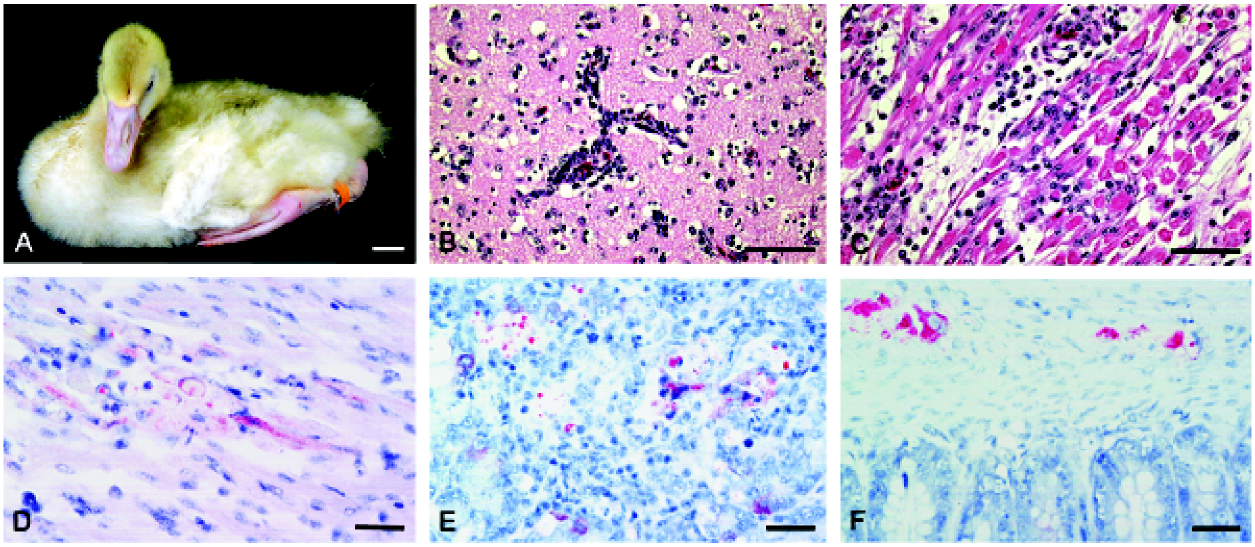

Figure. Two-week-old goslings that were subcutaneously inoculated with 9/99 New York strain of WN virus. (A) Abnormal posture of the head 10 days postinoculation (PI) (#80). The gosling had rhythmic side-to-side movement of the head. Bar = 2.5 cm. (B) Severe nonsuppurative encephalitis at 10 days PI (#80). HE stain. Bar = 50 µm. (C) Severe diffuse myocarditis with edema and myocyte necrosis at 6 days PI (#86). HE stain. Bar = 50 µm. (D) Flaviviral antigens in degenerating myocytes and macrophages within a focus of inflamed myocardium. Immunohistochemical (IHC) strain. Bar = 25 µm. (E) Flaviviral antigens in degenerating parenchymal epithelial cells and foamy macrophages within a focus of pancreatitis. IHC. Bar = 25 µm. (F) Flaviviral antigens in ganglion cells within muscularis mucosae. IHC. Bar = 25 µm.

inflammation of pecten oculi, the protruding vascular structure in the posterior vitreous; and heterophilic (purulent) scleritis and keratitis. WN virus was isolated from brain, heart, kidney, and intestine of goslings in the WN virus-inoculated group (Table). The virus titers were highest in #6, which died 5 days PI; intermediate in #86, which died 6 days PI; and lowest in #80, which was euthanized 10 days PI. WN virus was not isolated from any goslings sampled at the end of the experiment.

Immunohistochemical (IHC) assay, based on a two-step indirect immunoalkaline phosphatase technique described previously (11), was performed on multiple organs of goslings. The primary antibody used in the assay was a mouse polyclonal antibody that cross-reacts with several members of JE serocomplex group flavivirus, including JE virus, St. Louis encephalitis virus, and WN virus. Brain tissues from human JE- and WN virus-confirmed cases (positive controls) and from an unrelated human patient (negative control) were run in parallel. Immunostaining of flaviviral antigens was demonstrated in heart, brain, pancreas, kidney, and autonomic ganglion cells of the intestine (Figure) in WN virus-inoculated goslings, but the distribution and intensity varied with individual WN virus-inoculated goslings.

WN virus was isolated from plasma samples of WN virus-inoculated goslings collected between 1 and 5 days PI, with highest titers 2 and 3 days PI (Table). WN virus was isolated

Table. Virus isolation and titration data from 2-week-old domestic geese inoculated with WN virus and samples taken between 0 and 21 days postinoculation (PI)

| Group-bird | Plasma virus titers ($log_{10}$ $TCID_{50}$/0.1 mL)[a] by days PI | | | | | | | | | |
|---|---|---|---|---|---|---|---|---|---|---|
| identification | 0 | 1 | 2 | 3 | 4 | 5 | 7 | 10 | 14 | 21 |
| Sham controls: | | | | | | | | | | |
| 58 | - | NS | - | NS | - | NS | - | - | - | - |
| 73 | - | NS | - | NS | - | NS | - | - | - | - |
| WN virus-inoculates: | | | | | | | | | | |
| 6 | - | 1.5 | 5.5 | 4.5[b] | 2.5 | 2.5[c] | Dead | Dead | Dead | Dead |
| 78 | - | 3.5 | 4.5 | 3.5 | 1.5[b] | 1.5 | - | - | - | - |
| 80 | - | 2.5 | 5.5 | 2.5[b] | 1.5 | - | - | -[c] | Dead | Dead |
| 86 | - | 2.5 | 6.5 | 5.5 | 2.5 | 1.5[c] | Dead | Dead | Dead | Dead |
| In contacts: | | | | | | | | | | |
| 8 | - | NS | - | NS | - | NS | - | - | - | - |
| 13 | - | NS | - | NS | - | NS | - | 2.5 | - | - |

[a]Endpoints in Vero cell cultures based on CPE and confirmation as WN virus by indirect fluorescent antibody test: NS = No sample, - = No cytopathic effect.
[b]WN virus isolated from oropharyngeal swabs; #6, $10^{1.5}$/0.1 mL, #80, $10^{0.5}$/0.1 mL, #78, $10^{0.5}$/0.1 mL (reported as tissue culture infective doses [$TCID_{50}$] titers in oropharyngeal swab media; originally each swab placed in 1.5 mL of phosphate-buffered saline supplemented with 0.75% bovine serum albumin).
[c]WN virus isolated from tissues at necropsy ($TCID_{50}$/0.01 g): 1) Gosling #6 (5 days PI) - $10^{3.5}$ in brain, $10^{4.5}$ in heart, $10^{1.5}$ in kidney and $10^{3.5}$ in intestine; 2) gosling #80 (10 days PI) - $10^{1.5}$ in brain and $10^{1.5}$ in intestine; 3) gosling #86 (6 days PI) - $10^{3.5}$ in brain, $10^{2.5}$ in heart, $10^{1.5}$ in kidney and $10^{1.5}$ in intestine.

from oropharyngeal swabs on single sampling days in three of four WN virus-inoculated goslings but in low titers ($\leq 10^{1.5}$/0.1 mL). One in-contact gosling (an uninoculated gosling in contact with a WN virus-inoculated gosling) developed transient low-level viremia on 10 days PI (Table). No WN virus was isolated from cloacal swabs of WN virus-inoculated and in-contact goslings. Sham-control goslings were WN virus-free and negative on plaque reduction neutralization assay for anti-WN virus antibodies. WN virus-inoculated goslings had anti-WN virus antibodies 5,7,10, 14, and 21 days PI, and anti-WN virus antibodies were present in one in-contact gosling 14 and 21 days PI.

In the field, the incidence and outcome of WN virus infection vary with the host species. Wild birds are the primary reservoir hosts and, in WN virus-endemic areas, prevalence rates vary from 10% to 53% (12). Seroprevalence studies have identified natural infection in poultry species including broiler chickens and breeders, turkeys, breeder geese, and various species of pigeons in Israel, but veterinary medical investigations identified no associated disease or deaths except in pigeons (Y. Weisman, pers. comm.). However, WN virus infection has been associated with meningoencephalitis, clinical neurologic disease, and myocarditis in young domestic geese (6). The clinical and pathologic features of natural WN virus infections were reproduced in the current study. In humans, infections have usually been asymptomatic, but occasionally illness and death have resulted, accompanied by meningoencephalitis, anterior myelitis, acute pancreatitis, and myocarditis (12). During the 1999 New York outbreak of WN virus, histopathologic examination of central nervous system tissues from four fatal human cases showed varying degrees of neuronal necrosis, with infiltrates of microglia and polymorphonuclear leukocytes, perivascular cuffing, neuronal degeneration, and neuronophagia. IHC assay demonstrated immunostaining of flaviviral antigens in neurons, neuronal processes, and areas of necrosis. No immunostaining was seen in other major organs, including lung, liver, spleen, and kidney. The histopathologic lesions and immunostaining were more prominent in the brain stem and spinal cord (13). Similarly, various animal models, such as mice, hamsters, and rhesus monkeys, have developed fatal encephalitis upon intracerebral inoculation of WN virus (12). In natural cases of WN virus in zoologic birds representing 14 different species and eight orders, deaths were associated with severe myocarditis and encephalitis, and flaviviral antigen was demonstrated in cardiac myocytes and neurons (2,14). Findings from natural cases and experimental studies in various birds and mammals suggest common pathogenic effects of WN virus infections are caused by apoptotic or necrotic cell death in parenchymal cells of the brain, heart, and pancreas.

### Conclusions

In our study, subcutaneous inoculation with a New York WN virus isolate produced clinical signs of depression, weight loss, torticollis, opisthotonus, and death in 2-week-old domestic geese, similar to disease in reported field cases. Transient viremia developed 1 to 5 days PI with peak viremia titers ($10^{4-6}$) 2 days PI. Experimental studies in 1- to 11-day-old chicks demonstrated acquisition of infection from mosquitos, and the resulting viremia titers ($10^{4-6.3}$) efficiently infected naive mosquitoes (7). However, after 3 weeks of age, chickens were refractory to infection by mosquitos, and the resulting low viremia titers were inefficient in infecting mosquitoes (7). In contrast, the peak viremia titers ($10^{4-6}$) in goslings of the current study were of sufficient magnitude to efficiently infect mosquitos and serve as a reservoir and amplifying host. Furthermore, serologic and virologic data indicated that transmission of WN virus between goslings is possible without a mosquito vector, i.e., by direct contact. In contrast to geese, experimental WN virus infections did not produce clinical disease in chickens and turkeys, and the viremia levels were lower than occurred in the goslings (4,5). WN virus was not directly transmitted to in-contact chickens or turkeys. In addition to various wild birds species of the order Passiformes, young domestic geese can be a reservoir and amplifying host for WN virus and could contribute to the emerging ecology of WN virus in North America.

### Acknowledgments

We thank L. Perkins and L. Turpin for providing technical assistance and E. Ostlund and D. Senne for providing the challenge WN virus isolate and suggestions on laboratory assays.

Dr. Swayne is a veterinary pathologist and director of the Southeast Poultry Research Laboratory of the U.S. Department of Agriculture. His research focuses on pathobiology and control of exotic and emerging viral diseases of poultry and other birds, principally highly pathogenic avian influenza and West Nile virus.

### References

1. Komar N. West Nile viral encephalitis. Rev Sci Tech 2000;19:166-76.
2. Office International des Epizooties. West Nile fever in the United States of America: in horses. Disease Information 2000;13:150-1.
3. Rappole JH, Derrickson SR, Hubalek Z. Migratory birds and spread of West Nile virus in the Western hemisphere. Emerg Infect Dis 2000;6:319-28.
4. Swayne DE, Beck JR, Zaki SR. Pathogenicity of West Nile virus for turkeys. Avian Dis 2000;44:932-7.
5. Senne DA, Pedersen JC, Taylor WD, Hutto DL, Panigrahy B. Pathogenicity of West Nile virus for chickens. Avian Dis 2000;44:642-9.
6. Office International des Epizooties. West Nile fever in Israel in geese. Disease Information 1999;12:166.
7. Taylor RM, Work TH, Hurlbut HS, Rizk F. A study of ecology of the West Nile virus in Egypt. Am J Trop Med Hyg 1956;5:579-620.
8. Lanciotti RS, Roehrig JT, Deubel V, Smith J, Parker M, Steele K, et al. Origin of the West Nile virus responsible for an outbreak of encephalitis in the northeastern United States. Science 1999;286:2333-7.
9. Anderson JF, Andreadis TG, Vossbrinck CR, Tirrell S, Wakem EM, French RA, et al. Isolation of West Nile virus from mosquitoes, crows, and a Cooper's hawk in Connecticut. Science 1999;286:2331-3.
10. Barbeito MS, Abraham G, Best M, Cairns P, Langevin P, Sterritt WG, et al. Recommended biocontainment features for research and diagnostic facilities where animal pathogens are used. Rev Sci Tech 1995;14:873-7.
11. Zaki SR, Greer PW, Coffield LM, Goldsmith CS, Nolte KB, Foucar K, et al. Hantavirus pulmonary syndrome. Pathogenesis of an emerging infectious disease. Am J Pathol 1995;146:552-79.
12. Malik-Peiris JS, Amerasinghe FP. West Nile fever. In: Beran GW, Steele JH, editors. Handbook of Zoonoses Section B: Viral. Boca Raton: CRC Press; 1994. p. 139-48.
13. Shieh WJ, Guarner J, Layton M, Fine A, Miller J, Nash D, et al. The role of pathology in an investigation of an outbreak of West Nile encephalitis in New York, 1999. Emerg Infect Dis 2000;6:370-2.
14. Steele KE, Linn MJ, Schoepp RJ, Komar N, Geisbert TW, Manduca RM, et al. Pathology of fatal West Nile virus infections in native and exotic birds during the 1999 outbreak in New York City. Vet Pathol 2000;37:208-24.
